# Long-Chain Polyunsaturated Fatty Acids Accelerate the Rate of Insulin Aggregation and Enhance Toxicity of Insulin Aggregates

**DOI:** 10.1021/acschemneuro.3c00583

**Published:** 2023-12-21

**Authors:** Zachary Hoover, Michael Lynn, Kiryl Zhaliazka, Aidan P. Holman, Tianyi Dou, Dmitry Kurouski

**Affiliations:** † Department of Biochemistry and Biophysics, Texas A&M University, College Station, Texas 77843, United States; ‡ Department of Entomology, Texas A&M University, College Station, Texas 77843, United States; § Department of Biomedical Engineering, Texas A&M University, College Station, Texas 77843, United States

**Keywords:** LCPUFAs, LCUFAs, insulin, oligomers, fibrils, toxicity

## Abstract

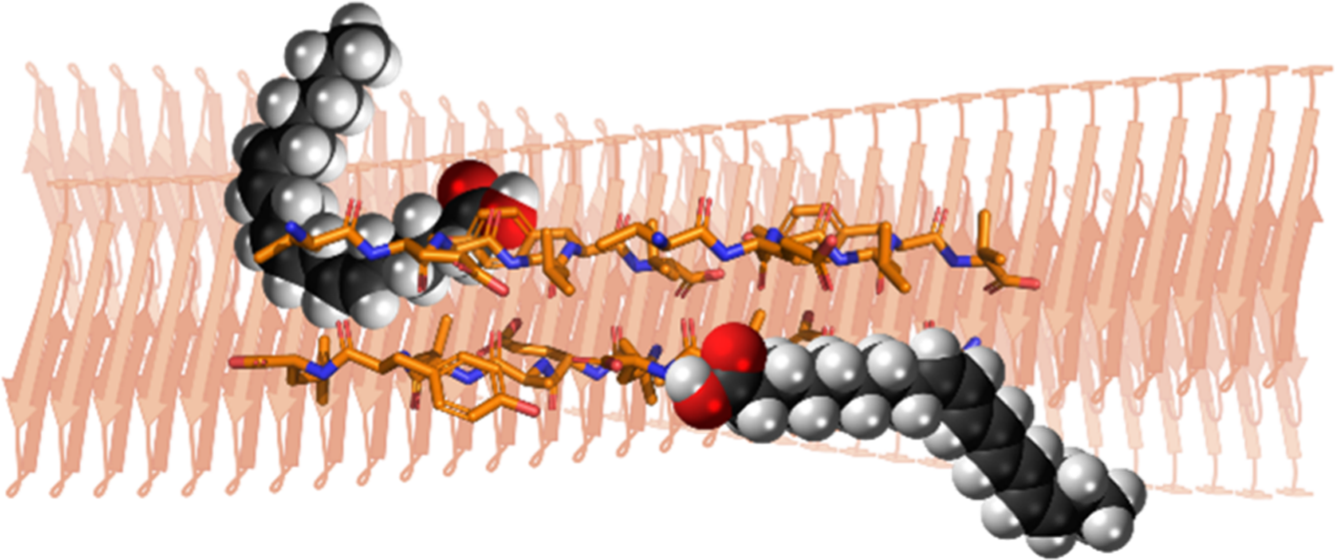

Long-chain polyunsaturated fatty acids (LCPUFAs) are essential components of a human diet. These molecules are critically important for cognitive attention and memory, mood states, coronary circulation, and cirrhosis. However, recently reported findings demonstrated that docosahexaenoic (DHA) and arachidonic acids (ARA), ω-3 and ω-6 LCPUFAs, accelerated the aggregation rates of insulin and α-synuclein, proteins that are directly linked to diabetes type 2 and Parkinson’s disease, respectively. Furthermore, both DHA and ARA uniquely altered the structure and toxicity of the corresponding protein aggregates. Our objective is to ascertain whether other LCPUFAs, alongside long-chain unsaturated fatty acid (LCUFA) proteins, exhibit similar effects on amyloidogenic proteins. To explore this matter, we investigated the effect of 10 different LCPUFAs and LCUFAs on the rate of insulin aggregation. We found that all of the analyzed fatty acids strongly accelerated insulin aggregation. Moreover, we found that protein aggregates that were formed in the presence of these fatty acids exerted significantly higher cell toxicity compared with insulin fibrils grown in the lipid-free environment. These findings show that interactions between amyloid-associated proteins and LCPUFAs can be the underlying molecular cause of neurodegenerative diseases.

## Introduction

Long-chain unsaturated and polyunsaturated fatty acids (LCUFAs and LCPUFAs) are a large group of fatty acids that play an important role in cell signaling, repair, and growth of the retina, neurons, and skeletal muscle tissue.^1,2^ These molecules constitute a substantial part of cell and organelle membranes.^3,4^ Therefore, LCUFAs and LCPUFAs are broadly utilized as food supplies. In the bloodstream, intercellular space, and cell cytosol, consumed LCUFAs and LCPUFAs interact with many molecules, including proteins and carbohydrates.^5,6^

Amyloid β (Aβ_1–42_) and α-synuclein (α-Syn) are directly linked to the onset and spread of Alzheimer’s and Parkinson’s diseases. Galvagnion and co-workers found that lipids could change the rate of α-Syn aggregation.^7−9^ Specifically, in the presence of large unilamellar vesicles of phospholipids, α-Syn exhibited significantly greater rates of aggregation compared to those in the lipid-free environment. However, with an increase in the concentration of lipid vesicles relative to the concentration of the protein, the rate of α-Syn aggregation was lowered.^7−9^ These findings suggest that an increase in the lipid bilayer surface decreased the probability of protein–protein interactions that are critically important for amyloid fibril formation. Dou and co-workers found that the lipids phosphatidylcholine (PC) and phosphatidylserine (PS) not only altered the rate of α-Syn aggregation but also uniquely modified the secondary structure of α-Syn protein oligomers.^10,11^ Expanding upon this, Matveyenka and co-workers demonstrated that lipids could also alter the rates of insulin aggregation, as well as modify the secondary structure of both oligomers and fibrils formed in their presence.^12−17^ Furthermore, protein aggregates that were formed in the presence of lipids exerted significantly lower cell toxicity compared to the protein fibrils formed in the lipid-free environment.^12−17^ Recently reported results by Zhaliazka and co-workers demonstrated that similar conclusions could be made about lysozyme and Aβ_1–42_ aggregation.^18,19^ Specifically, it was found that PC, cholesterol, and cardiolipin accelerated the rate of Aβ_1–42_ aggregation and changed the secondary structure of β_1–42_ oligomers and fibrils.^18^ This resulted in a drastic increase in the toxicity of such aggregates compared to those formed in the lipid-free environment.

The key inquiry revolving around LCUFAs and LCPUFAs is whether these molecular species can alter the rate of insulin aggregation and modify the toxicity of insulin aggregates. Insulin is a small hormone that regulates glucose metabolism. Its aggregation can cause diabetes type 2 and injection amyloidosis.^20,21^ In the former case, an overproduction of insulin significantly increases the hormone concentration in the pancreas.^22^ This can trigger protein aggregation, which results in the formation of highly toxic oligomers and fibrils.^23,24^ In the latter case, similar protein aggregates can be formed in the derma as a result of insulin injection.^25^ In both cases, insulin oligomers and fibrils may trigger the aggregation of other proteins, which results in systemic amyloidosis.^26^

In this study, we investigated the effect of LCUFAs that had 16 and 18 carbon atoms and one double bond in *cis* and *trans* configurations as well as LCPUFAs with 2, 3, 4, 5, and 6 double bonds that possessed 18 and 20 carbon atoms on the rate of insulin aggregation. We also used atomic force microscopy (AFM) to reveal the extent to which LCUFAs and LCPUFAs alter the morphology of insulin aggregates, as well as infrared (IR) spectroscopy and circular dichroism (CD) to investigate the secondary structure of insulin fibrils formed in the presence of LCUFAs and LCPUFAs. Finally, we employed a set of molecular biology assays to determine the toxicity of insulin aggregates.

## Results and Discussion

### Elucidation of the Rate of Insulin Aggregation in the Presence of LCUFAs and LCPUFAs

**Figure 1:**
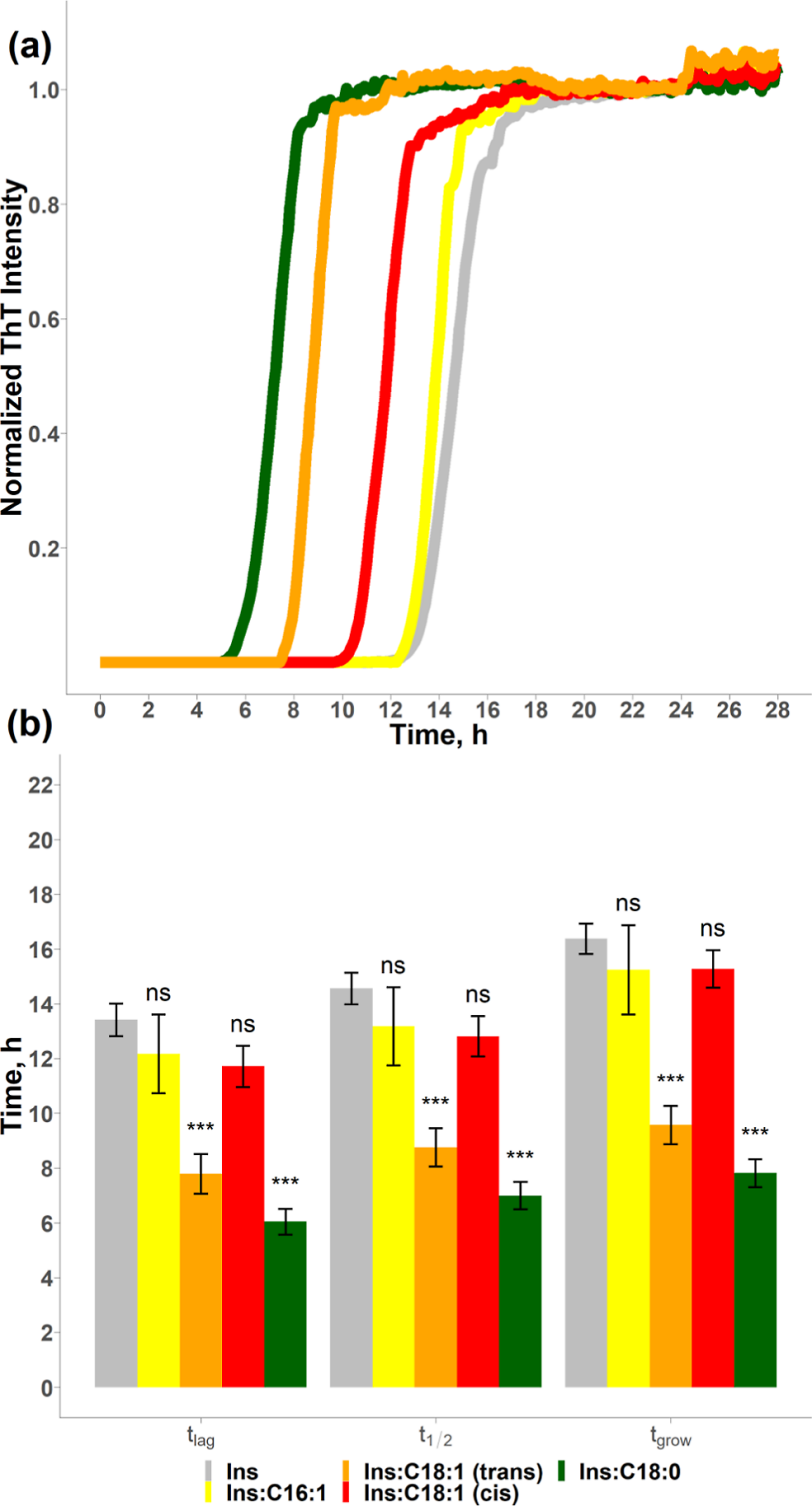
LCUFAs uniquely alter the aggregation rate of insulin. ThT aggregation kinetics (a) with a histogram (b) that summarizes the mean values (±SEM) of *t*_lag_, *t*_1/2_, and *t*_grow_ of insulin in the lipid-free environment (gray), as well as in the presence of C16:1 (yellow), C18:0 (green), *trans* C18:1 (orange), and *cis* C18:1 (red). *P* < 0.1, *P < 0.05, **P < 0.01, ***P < 0.001. “NS” nonsignificant difference. Each kinetic curve is the average of at least three independent measurements.

In the lipid-free environment, insulin (Ins) aggregates exhibit a well-defined lag phase (*t*_lag_ = 13.4 ± 0.6 h) that is characterized by a progressive accumulation of protein oligomers, Figure 1. Once a critical value of their concentration is reached, oligomers rapidly propagate into fibrils, protein aggregates that bind thioflavin T (ThT). As a result of this binding, the quantum yield of the ThT fluorescence drastically increases. This phenomenon can be used for *in situ* monitoring of protein aggregation, as well as a rough quantification of the number of fibrillar species in the analyzed samples.

**Figure 2:**
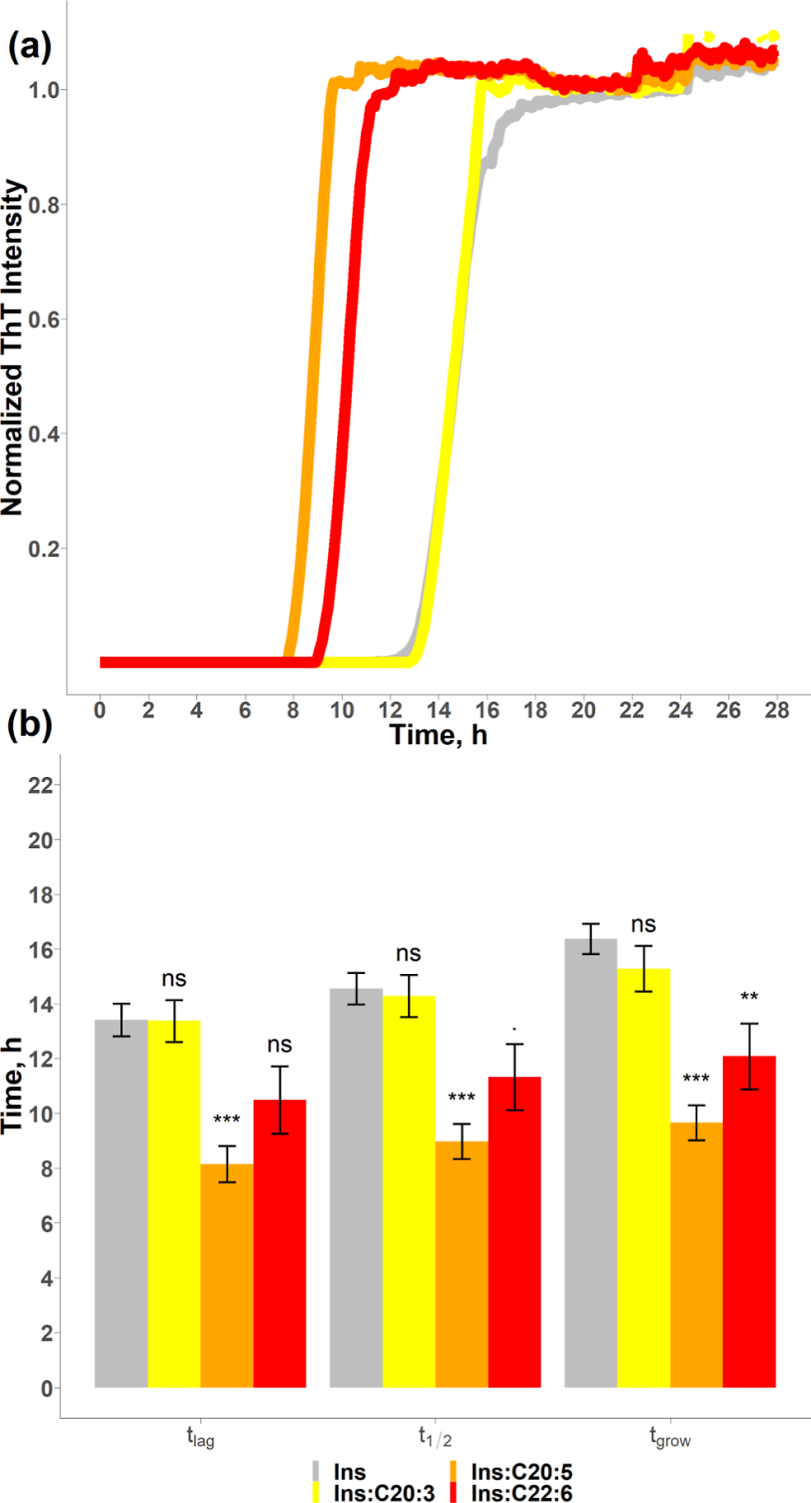
LCPUFAs uniquely alter the aggregation rate of insulin. ThT aggregation kinetics (a) with a histogram (b) that summarizes the mean values (±SEM) of *t*_lag_, *t*_1/2_, and *t*_grow_ of insulin in the lipid-free environment (gray), as well as in the presence of C20:3 (yellow), C20:5 (orange), and C22:6 (red). *P* < 0.1, *P < 0.05, **P < 0.01, ***P < 0.001. “NS” nonsignificant difference. Each kinetic curve is the average of at least three independent measurements.

We found that the *trans* C18:1 LCUFA (*t*_lag_ = 7.8 ± 0.7 h) shortened the lag phase of protein aggregation while the corresponding *cis* C18:1 LCUFA (*t*_lag_ = 11.7 ± 0.7 h) did not significantly shorten the lag phase, Figure 1. Based on this result, we can conclude that *trans* LCUFAs cause a much stronger shortening of the lag phase than their *cis* analogues. Our findings showed that a fully saturated analogue of both *cis* and *trans* C18:1 LCUFA, C18:0 LCUFA also shortened the lag phase of insulin aggregation (*t*_lag_ = 6.0 ± 0.5 h), Figure 1. These findings suggest that a hydrophobic nature of LCUFAs micelles rather than double bonds themselves could trigger protein aggregation, shortening the lag phase of insulin fibril formation. However, C16:1 LCUFA (*cis*) did not alter the rate of insulin aggregation (*t*_lag_ = 12.2 ± 1.4 h), Figure 1. However, similar to other LCUFAs, C16:1 formed micelles. Thus, one can expect that the length of the FA tail can be the key determinant in the reduction of the lag phase of protein aggregation. To test this hypothesis, we determined the lag phase of insulin aggregation in the equimolar presence of C20 and C22 LCPUFAs with 3, 5, and 6 double bonds, Figure 2.

**Figure 3:**
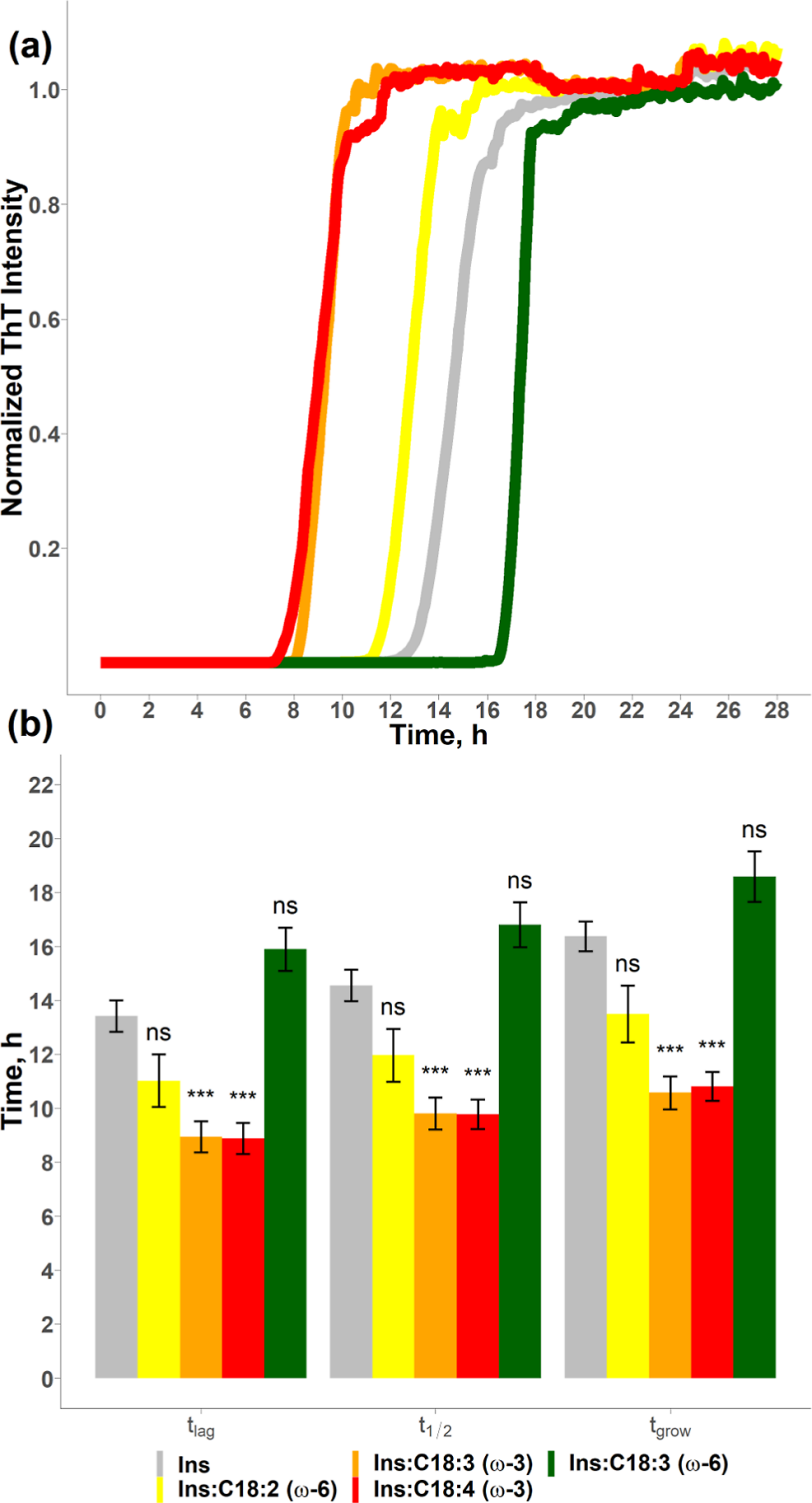
LCPUFAs uniquely alter the aggregation rate of insulin. ThT aggregation kinetics (a) with a histogram (b) that summarizes the mean values (±SEM) of *t*_lag_, *t*_1/2_, and *t*_grow_ of insulin in the lipid-free environment (gray), as well as in the presence of C18:2 (yellow), C18:3 (orange), C18:4 (red), and C18:3 (green). *P* < 0.1, *P < 0.05, **P < 0.01, ***P < 0.001. “NS” nonsignificant difference. Each kinetic curve is the average of at least three independent measurements.

We found that one of these C20 and C22 FAs was able to shorten the lag phase of insulin aggregation reducing *t*_lag_ to 8.2 ± 0.7 h (C20:5), Figure 2. However, C20:3 (*t*_lag_ = 13.4 ± 0.8 h) and C22:6 (*t*_lag_ = 10.5 ± 1.2 h) did not significantly alter the *t*_lag_ of insulin aggregation. It should be noted that C20:5 is an ω-3 LCPUFA, whereas C20:3 is an ω-6 LCPUFA. Thus, the localization of the unsaturated bonds rather than the length of the carbon chain of LCPUFAs could be a major determinant of the lag phase of insulin aggregation. To test this hypothesis, we analyzed the effect of ω-6 and ω-3 LCPUFAs with 18 carbon atoms, as shown in Figure 3.

We found that ω-3 LCPUFAs (C18:3 and C18:4) were able to demonstrate the shortest lag phase if added to insulin in equimolar concentrations, with both LCPUFAs reducing *t*_lag_ to 8.9 ± 0.6 h, Figure 3. At the same time, the presence of ω-6 C18:2 (*t*_lag_ = 11.0 ± 1.0 h) did not cause a significant change in the lag phase of protein aggregation (*t*_lag_ for insulin was 13.4 ± 0.6 h), Figure 3. We also found that ω-6 C18:3 (*t*_lag_ = 15.9 ± 0.8 h) did not significantly affect *t*_lag_. These results confirmed that localization of the last double bond in the FA chain (ω-3 *vs* ω-6) and not the number of double bonds themselves is a critically important factor that determines the lag phase of protein aggregation. Finally, our results showed that conformation of the double bonds (*cis vs trans*) was a critically important property of FAs in terms of the lag phase of insulin aggregation. It should be noted that FAs in living organisms have predominantly a *cis* configuration of double bonds. Thus, the “natural” configuration of the double bond in LCUFAs has a weaker effect on the rate of insulin aggregation compared to the *trans* configuration that can be found in “synthetic” fats.

We also found that in addition to shortening the lag phase of insulin aggregation, LCUFAs and LCPUFAs uniquely altered the rate of fibril formation. In the lipid-free environment, insulin exhibits a *t*_1/2_ of 14.6 ± 0.6, Figure 1. However, in the presence of *trans* C18:1 LCUFA, *t*_1/2_ was reduced to 8.8 ± 0.7 h. At the same time, C16:1 (*t*_1/2_ = 13.2 ± 1.4 h) and *cis* C18:1 (*t*_1/2_ = 12.8 ± 0.7 h) demonstrated only insignificantly different *t*_1/2_ than insulin itself, Figure 1. C18:0 FA exhibited a similar acceleration rate of insulin aggregation as *trans* C18:1 LCUFA (8.8 ± 0.7 h). We also found that all analyzed ω-3 LCPUFAs strongly accelerated the rate of insulin aggregation reducing *t*_1/2_ from 14.6 ± 0.6 h (insulin) to 9.8 ± 0.6 h (C18:3), 11.3 ± 1.2 h (C22:6), 9.0 ± 0.6 h (C20:5), and 9.8 ± 0.5 h (C18:4), Figures 2 and 3. At the same time, no change in the rate of insulin aggregation was observed in the presence of equimolar concentrations of ω-6 LCPUFAs. Thus, we can conclude that ω-3 LCPUFAs, as well as *trans* FAs, strongly accelerated the rate of insulin aggregation.

### Morphological Examination of Insulin Aggregates Grown in the Presence of LCUFAs and LCPUFAs

**Figure 4:**
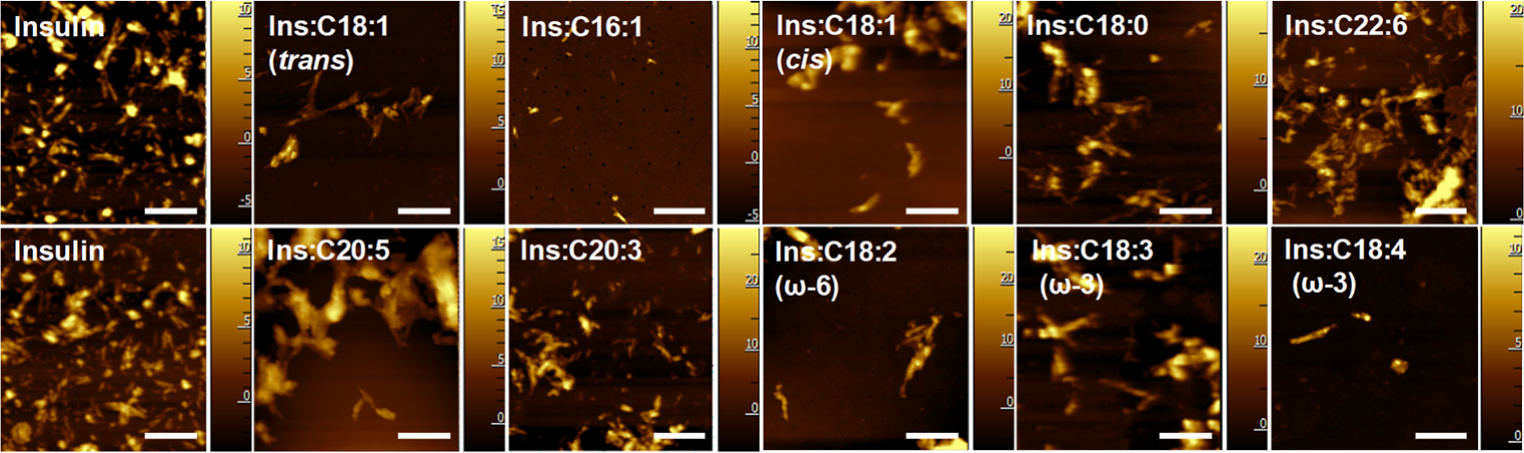
Morphology of insulin aggregates grown in a lipid-free environment (Ins) and in the presence of LCUFAs and LCPUFAs. White scale bars are 500 nm. *Z* scale bars are in nm.

Microscopic examination of insulin aggregates formed in the lipid-free environment (Ins) revealed the presence of fibril species that were 10–12 nm in height (Figures 4 and S1). Morphologically similar aggregates were observed in all other samples, as shown in Figure 4. However, in most of them, except Ins:C16:1 and Ins:C18:4 (ω-3), we observed an accumulation of lipid shells around the fibril species. This resulted in a substantial increase in the height (10–20 nm) of these aggregates. Such lipid–fibril clusters dominate in Ins:C18:1 (*trans*), Ins:C22:6, Ins:C20:5, and Ins:C18:3 (ω-3) but were less abundant in Ins:C18:1 (*cis*), Ins:C18:0, Ins:C20:3, and Ins:C18:2 (ω-6). These findings demonstrate that LCUFAs and LCPUFAs tend to accumulate on the surface of insulin aggregates formed in their presence. At the same time, our results suggest that both LCUFAs and LCPUFAs cause little to no change in the topology of insulin fibrils themselves.

### Revealing the Secondary Structure of Insulin Aggregates Grown in the Presence of LCUFAs and LCPUFAs

IR spectra acquired from protein aggregates grown in the presence of LCUFAs and LCPUFAs, as well as insulin fibrils formed in the lipid-free environment, exhibit both amide I (1600–1700 cm^–1^) and II (1500–1550 cm^–1^) vibrational bands, Figure S1. In all acquired spectra, the amide I band has a strong peak at 1630 cm^–1^ with a shoulder at ∼1660 cm^–1^, which indicates the dominance of parallel β-sheets in the secondary structure of these aggregates with a small amount of unordered protein secondary structures, Figure S2. It should be noted that IR did not reveal any significant differences in the secondary structures of LCUFAs and LCPUFAs compared to the fibrils formed by insulin in the lipid-free environment. Similar conclusions could be made based on the CD spectra acquired from these samples. We found that all CD spectra exhibit a trough at ∼220 nm, which also indicates the presence of a parallel β-sheet in the secondary structure of these aggregates, Figure S3. Thus, we can conclude that protein aggregates grown in the presence of LCUFAs and LCPUFAs, as well as insulin fibrils formed in the lipid-free environment, have a parallel β-sheet secondary structure.

**Figure 5:**
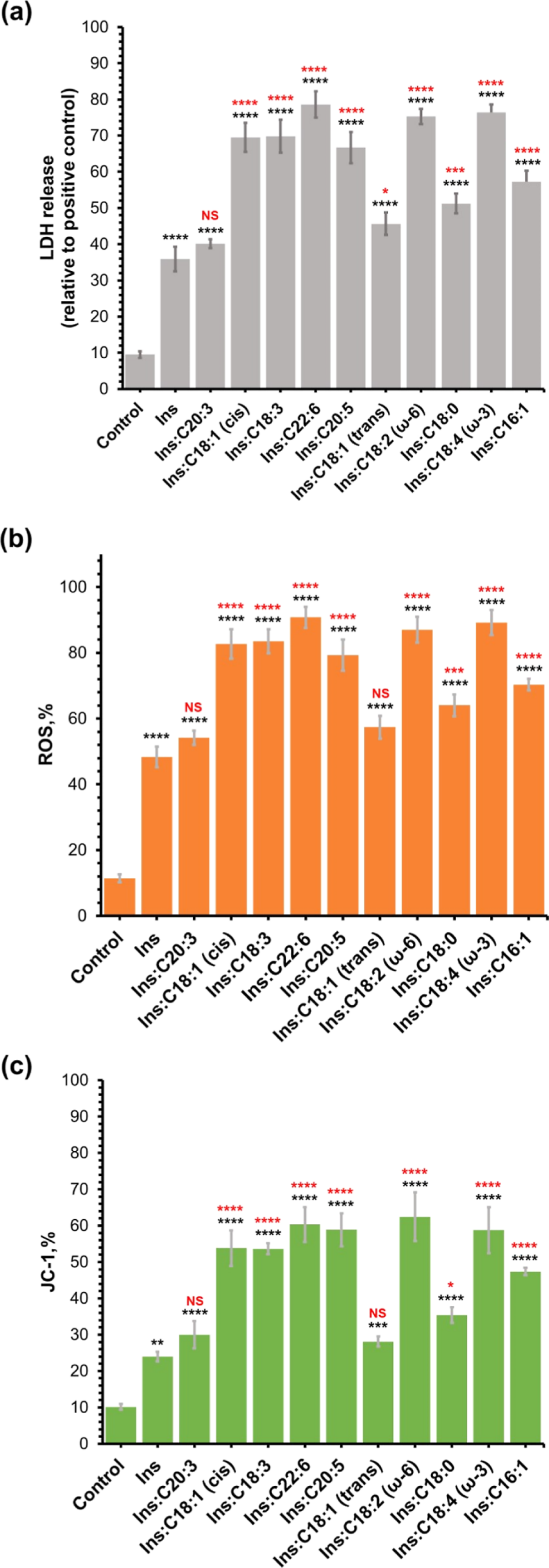
Insulin aggregates grown in the presence of LCUFAs and LCPUFAs exert different cell toxicity compared to that of the aggregates grown in the LCPUFA-free environment. Histograms of LDH (a), ROS (b), and JC-1 (c) assays of insulin aggregates grown in the presence of LCUFAs and LCPUFAs, as well as in the lipid-free environment (Ins). Black asterisks (*) show a significance level of differences between protein aggregates and the control; red asterisks (*) show a significance level of differences between Ins and protein aggregates grown in the presence of LCUFAs and LCPUFAs. *P < 0.05, **P < 0.01, ***P < 0.001, ****P < 0.0001. “NS” nonsignificant difference.

Whether the presence of LCUFAs and LCPUFAs alters the toxicity of insulin fibrils formed in their presence is a crucial inquiry. To address this, we utilized a mouse midbrain N27 cell line and conducted a series of toxicity assays, revealing the toxicity of Ins:LCUFA and Ins:LCPUFA fibrils, Figure 5.

We found that all Ins:LCUFA and Ins:LCPUFA fibrils except Ins:C20:3 exerted significantly higher cell toxicity compared to the toxicity of Ins fibrils formed in the lipid-free environment. Furthermore, ω-3 C18:3, ω-3 C18:4, ω-6 C18:2, and C22:6 exerted the highest cell toxicity compared to all other Ins:LCUFA and Ins:LCPUFA aggregates. We also found that Ins:C18:1 (*cis*) fibrils were found to be more toxic compared to Ins:C18:1 (*trans*) aggregates. Finally, the toxicity of Ins:C18:1 (*cis*) fibrils was similar to that exerted by Ins:C20:5 fibrils. These results demonstrated that LCUFAs and LCPUFAs uniquely altered the toxicity of insulin aggregates. It should be noted that LCUFAs and LCPUFAs themselves were not toxic to N27 rat neuronal cells (Figure S3).

Previously reported results by our and other research groups showed that amyloid aggregates enhance reactive oxygen species (ROS) production and cause mitochondrial dysfunction in cells.^27,28^ Therefore, we investigated the extent to which Ins:LCUFA and Ins:LCPUFA fibrils were engaged in ROS production and mitochondrial dysfunction of rat neuronal N27 cells, Figure 5. Our results showed that all Ins:LCUFA and Ins:LCPUFA fibrils except Ins:C20:3 exerted significantly higher levels of ROS and caused the strongest mitochondrial dysfunction in cells. We also found that cells exposed to ω-3 C18:3, ω-3 C18:4, ω-6 C18:2, and C22:6 exhibited the highest levels of ROS and mitochondrial dysfunction compared with the cells exposed to all other Ins:LCUFA and Ins:LCPUFA aggregates. Our findings demonstrate that Ins:C18:1 (*cis*) fibrils exert higher levels of ROS simultaneously causing the strongest mitochondrial dysfunction compared to Ins:C18:1 (*trans*) aggregates. These results showed that LCUFAs and LCPUFAs uniquely altered ROS levels and the magnitude of mitochondrial dysfunction that the corresponding insulin aggregates exert in N27 cells. It should be noted that LCUFAs and LCPUFAs themselves did not exert statistically significant ROS or JC-1 response in N27 rat neuronal cells, Figure S4.

Our results showed that LCUFAs and LCPUFAs strongly alter the aggregation rate of insulin. We found that most of the analyzed UFAs accelerated protein aggregation. This effect is likely linked to the localization of the distal double bond (ω-3 *versus* ω-6) in the UFAs. Specifically, all ω-3 LCPUFAs strongly accelerated protein aggregation, whereas ω-6 and other LCUFAs and LCPUFAs could or could not exert similar effects on the rate of protein aggregation. Using molecular docking simulations, Holman and co-workers recently showed that the ω-3 C18:3 LCPUFA has much lower binding energy to insulin compared to C22:6, C20:5, C16:1, C18:0, and C18:1 LCPUFAs and LCUFAs.^29^ Although no calculations were made for C18:4 (ω-3 LCPUFAs) and C18:2 (ω-6 LCPUFAs), these results indicate that observed differences in the rate of protein aggregation could be linked to the more favorable interaction of ω-3 LCPUFAs with insulin.

Microscopic examination of protein aggregates formed in the presence of LCUFAs and LCPUFAs revealed the presence of fibril species that had similar dimensions to the protein aggregates formed in the UFA-free environment. However, we found that these aggregates tend to accumulate LCUFAs and LCPUFAs on their surface. Previously reported structural analysis of Ins:arachidonic acid (ARA) and Ins:docosahexaenoic acid (DHA) fibrils using AFM-IR revealed the presence of the corresponding LCPUFAs in their structure.^30^ Furthermore, it was found that both ARA and DHA uniquely altered the secondary structure of such insulin aggregates. Similar results were reported by Matveyenka and co-workers for α-Syn. Specifically, the researchers found that both ARA and DHA strongly accelerated the rate of α-Syn aggregation. Using NMR and TEM, Lücke demonstrated that FAs strongly interact with α-Syn, whereas De Franceschi and Polverino de Laureto found that such interactions were primarily determined by 2–60 amino acids located in the N-termini of α-Syn.^31,32^ Protein:FA interactions play a critically important role in α-Syn aggregation. For instance, an α-Syn mutant with the deleted N-terminus, essential for FA binding, was not able to oligomerize.^33^ At the same time, α-Syn oligomers formed in the presence of LCPUFAs exhibited drastically different secondary structures compared to the oligomers formed in the FA-free environment.^30,34,35^

Localization of LCUFAs and LCPUFAs on the surface of protein aggregates helps to explain their superior toxicities to the N27 cell line compared to the toxicity of insulin fibrils formed in the UFA-free environment.^30^ One can expect that the lipid shell around insulin fibrils facilitates the permeability of these aggregates across the plasma membranes. As a result, the toxicity of such protein–UFA aggregates was found to be significantly greater than the toxicity of insulin fibrils formed in the UFA-free environment. We also found that aggregates which possessed *cis* LCUFAs exerted higher cell toxicity compared to fibrils formed in the presence of *trans* analogues of such LCUFAs. Finally, our results showed that insulin fibrils formed in the presence of ω-3 LCPUFAs exerted the strongest cell toxicity compared with insulin aggregates grown in the presence of other LCUFAs and LCPUFAs.

## Methods

### Materials

Bovine insulin was purchased from Sigma-Aldrich (St. Louis, MO, USA), and we utilized a variety of fatty acids, including eicosapentaenoic acid (EPA, 5*Z*,8*Z*,11*Z*,14*Z*,17*Z*-eicosapentaenoic acid, Calbiochem Cat: 324875-25MG) C20:5, DHA (4*Z*,7*Z*,10*Z*,13*Z*,16*Z*,19*Z*-docosahexaenoic acid, Acros Organics CAS: 6217-54-5) C22:6, α-linolenic acid (ALA, 9*Z*,12*Z*,15*Z*-α-linolenic acid, Acros Organics CAS: 463-40-1) C18:3, dihomo-γ-linolenic acid (DGLA, 8*Z*,11*Z*,14*Z*-dihomo-γ-linolenic acid, Enzo Cat no. BML-FA009-0100) C20:3, stearidonic acid (SDA, 4*Z*,7*Z*,11*Z*,13*Z*-eicosatetraenoic acid, Cayman Chemical Company Item: 90320) C18:4, stearic acid (STA, octadecanoic acid, Ward’s Science+ CAS: 57-11-4) C18:0, linoleic acid (LA, 9*Z*,12*Z*-octadecadienoic acid, EMD Millipore Corp. CAS: 436305-5GM) C18:2, *cis*-vaccenic acid (*cis*) [VA, (11*Z*)-octadec-11-enoic acid, Indofine Chemical Company CAS: 506-17-2] C18:1, elaidic acid (*trans*) [EA, (*E*)-octadec-9-enoic acid, Alfa Aesar CAS: 112-79-8] C18:1, palmitoleic acid (PA, 9-hexadecenoic acid, MP Biomedicals CAS: 373-49-9) C16:1, and γ-linolenic acid (GLA).

### FA Stock Preparation

Each fatty acid was weighed/aliquoted and dissolved/diluted to 40 mM using PB. Diluted samples were placed in an ultrasonic water bath for 30–60 min (50 °C). Samples were periodically vortexed every 5–10 min. Once the FAs were dissolved, samples were diluted to a working concentration of 400 μM by using PB. 40 mM stocks were stored at −20 °C at pH 7. We used 40 mM LCUFAs and LCPUFAs in all experiments, Table S1.

### Insulin Aggregation

In the UFA-free environment, 400 μM insulin was dissolved in PBS. After that, the pH of the protein solution was adjusted to pH 3.0 using concentrated HCl. For LCUFA and LCPUFA samples, 400 μM of insulin was mixed with an equivalent concentration of the corresponding LCUFAs and LCPUFAs. Next, we adjusted the pH of the final solution to pH 3.0 using concentrated HCl. Finally, protein samples were added to a 96-well plate and kept in a plate reader (Tecan, Männedorf, Switzerland) at 37 °C for 28 h under 510 rpm agitation.

### Kinetic Measurements

The rates of insulin aggregation were measured using a ThT fluorescence assay. For this, samples were mixed with a ThT solution to reach a final ThT concentration of 30 μM. Samples were added into a 96-well plate and kept in a plate reader (Tecan, Männedorf, Switzerland) at 37 °C for 28 h under 510 rpm agitation. Fluorescence measurements were taken every 10 min (excitation, 450 nm; emission, 495 nm).

### AFM Imaging

Microscopic analysis of protein aggregates was performed on an AIST-NT-HORIBA system (Edison, NJ). We used silicon AFM probes (force constant 2.7 N/m; resonance frequency 50–80 kHz) that were purchased from AppNano (Mountain View, CA, USA). For each sample, an aliquot of the protein sample was diluted 10–50 times with DI water and placed onto a precleaned glass coverslip. After 5–24 h of exposure, the surface of the slide was rinsed with DI water and dried under a dry air flow. Image preprocessing and analysis were done using AIST-NT software (Edison, NJ, USA).

### Circular Dichroism

For all measurements, 300 μL of the protein aggregate was collected and added into a 1 mm quartz cuvette. CD spectra were acquired on a J-1000 CD spectrometer (Jasco, Easton, MD, USA). Three spectra were collected for each sample within 195–250 nm and averaged using Thermo Grams Suite software (Thermo Fisher Scientific, Waltham, MA, USA).

### Attenuated Total Reflectance Fourier-Transform Infrared (ATR-FTIR) Spectroscopy

An aliquot of the protein sample was placed on an ATR crystal and dried at room temperature. The spectra were measured by using a Spectrum 100 FTIR spectrometer (PerkinElmer, Waltham, MA, USA). Three spectra were collected from each sample and averaged using Thermo Grams Suite software (Thermo Fisher Scientific, Waltham, MA, USA).

### Cell Toxicity Assays

Rat midbrain N27 cells were grown in RPMI 1640 medium (Thermo Fisher Scientific, Waltham, MA, USA) with 10% fetal bovine serum (FBS) (Invitrogen, Waltham, MA, USA) in a 96-well plate (10,000 cells per well) at 37 °C under 5% CO_2_. After 24 h, the cells were found to fully adhere to the wells reaching ∼70% confluency. Next, 100 μL of the cell culture was replaced with 100 μL of RPMI 1640 medium with 5% FBS-containing and 10 μL of protein samples. After 24 h of incubation with the sample of the protein aggregates, a lactate dehydrogenase (LDH) assay was performed on the cell medium using a CytoTox 96 nonradioactive cytotoxicity assay (G1781, Promega, Madison, WI, USA). Absorption measurements were taken in a plate reader (Tecan, Männedorf, Switzerland) at 490 nm.

In parallel, an ROS assay was performed by using the same cell culture. Briefly, ROS reagent (C10422, Invitrogen, Waltham, MA, USA) was added to reach the final concentration of 5 μM and the mixture was incubated at 37 °C under 5% CO_2_ for 30 min. After the supernatant was removed, cells were washed with PBS and resuspended in 200 μL of PBS in flow cytometry tubes. Sample measurements were made in an Accuri C6 flow cytometer (BD, San Jose, CA, USA) using a red channel (λ = 633 nm). Data was analyzed using Acura software. The percentage of ROS production was calculated relative to the positive control in which cells were incubated with menadione at a final concentration of 200 μM for 30 min.

For JC-1 staining, JC-1 reagent (M34152A, Invitrogen) was added to the cells to achieve a final concentration of 50 μM and incubated at 37 °C in a 5% CO_2_ environment for 30 min. After the supernatant was removed and the cells were treated with trypsin, they were resuspended in 200 μL of 1× PBS at pH 7.4. Sample measurements were obtained using the green channel (λ = 488 nm) of an Accuri C6 flow cytometer (BD, San Jose, CA, USA). Data was analyzed using Acura software. The percentage of mitochondrial membrane depolarization was calculated relative to the positive control, where cells were incubated with carbonyl cyanide chlorophenylhydrazone for 5 min at a final concentration of 50 μM. All measurements were conducted in triplicates.

### Data Analysis

#### Kinetics Data Analysis

Individual aggregation replicates that did not aggregate within the observed time (between 22 and 48 h) were manually removed. Background ThT fluorescence, which was taken as the average of 3 wells for each experiment, was subtracted from each time point of the remaining replicates. Next, for each replicate, fluorescence intensities were normalized with the final intensity measured set to 1. Kinetics curves display the median normalized fluorescence intensity of all replicates of each indicated treatment.

In the reported results, *t*_1/2_ for each replicate was taken as the time point with the measured fluorescence intensity closest to 50% maximum intensity; *t*_lag_ for each replicate was determined as the time point with the fluorescence intensity closest to 10% maximum intensity. Finally, *t*_grow_ for each replicate was calculated as the time point with the fluorescence intensity closest to 90% maximum intensity.

Bar graphs display the average *t*_lag_, *t*_1/2_, and *t*_grow_ values for each treatment. Error bars display the standard error of the mean for *t*_lag_, *t*_1/2_, and *t*_grow_ values for each treatment.

The significance value for each Ins:FA treatment is the result of a one-way analysis of variance (ANOVA) with posthoc Tukey’s honest significant difference (HSD) test between that Ins:FA treatment and the Ins control. Data processing, statistical tests, and plots for kinetics were done in R v4.2.2, with the following packages:

dplyr v1.1.1
ggplot2 v3.4.2
gridExtra v2.3
gtools v3.9.4
Hmisc v5.1-1

### Cell Toxicity

For the evaluation of cytotoxicity assays, statistical analysis was conducted using MATLAB software. A one-way ANOVA was employed to determine the significance of the data, with a threshold of *p* < 0.05 considered indicative of statistical significance. A posthoc Tukey’s HSD multiple comparison test was utilized to further dissect the differences between groups. The results from these statistical tests were graphically represented in figures using Microsoft Excel.

## Supporting Information Available

Histograms of length distributions of Ins fibrils and fibrils formed in the presence of FAs, FTIR and CD spectra, histograms of LDH, ROS, and JC1 results of LCUFAs and LCPUFAs, and CMC of FAs used in the study (PDF)
