# Supplementary material for: Long-Chain Polyunsaturated Fatty Acids Accelerate the Rate of Insulin Aggregation and Enhance Toxicity of Insulin Aggregates
Source: ACS Chem Neurosci. 2023 Dec 21;15(1):147–54. doi: 10.1021/acschemneuro.3c00583 (PMC10862472; doi:10.1021/acschemneuro.3c00583)
Supplement: Supplementary file 1 — cn3c00583_si_001.pdf [file cn3c00583_si_001.pdf]

Long-Chain Polyunsaturated Fatty Acids Accelerate the Rate of Insulin Aggregation and Enhance Toxicity of Insulin Aggregates

Zachary Hoover[1‡], Michael Lynn[1‡], Kiryl Zhaliazka[1], Aidan Holman[2], Tianyi Dou[1] and Dmitry Kurouski[*1,3]

1. Department of Biochemistry and Biophysics, Texas A&M University, College Station, Texas 77843, United States
2. Department of Entomology, Texas A&M University, College Station, Texas 77843, United States
3. Department of Biomedical Engineering, Texas A&M University, College Station, Texas, 77843, United States

Supporting Information

Table S1. CMC for FAs used in the experiments.

FA	CMC	experimental	Reference
DHA	60 uM	400 uM	https://doi.org/10.1002%2Fj.1460-2075.1991.tb07651.x
ALA	1 mM	400 uM	https://doi.org/10.3390/min10100905
DGLA	0.35 mM	400 uM	https://www.govinfo.gov/content/pkg/GOVPUB-C13-88e516148b54d2428c9aa5befc7ab145/pdf/GOVPUB-C13-88e516148b54d2428c9aa5befc7ab145.pdf
SDA	1.4 mM	400 uM	https://www.govinfo.gov/content/pkg/GOVPUB-C13-88e516148b54d2428c9aa5befc7ab145/pdf/GOVPUB-C13-88e516148b54d2428c9aa5befc7ab145.pdf
STA	100 uM	400 uM	https://www.mdpi.com/2075-163X/10/10/905#
LA	60 uM	400 uM	https://doi.org/10.1002%2Fj.1460-2075.1991.tb07651.x
VA	700 uM	400 uM	https://doi.org/10.1079/BJN19690032
EA	2.5 mM	400 uM	https://doi.org/10.1246/bcsj.31.467
PA	100 uM	400 uM	https://doi.org/10.1002%2Fj.1460-2075.1991.tb07651.x

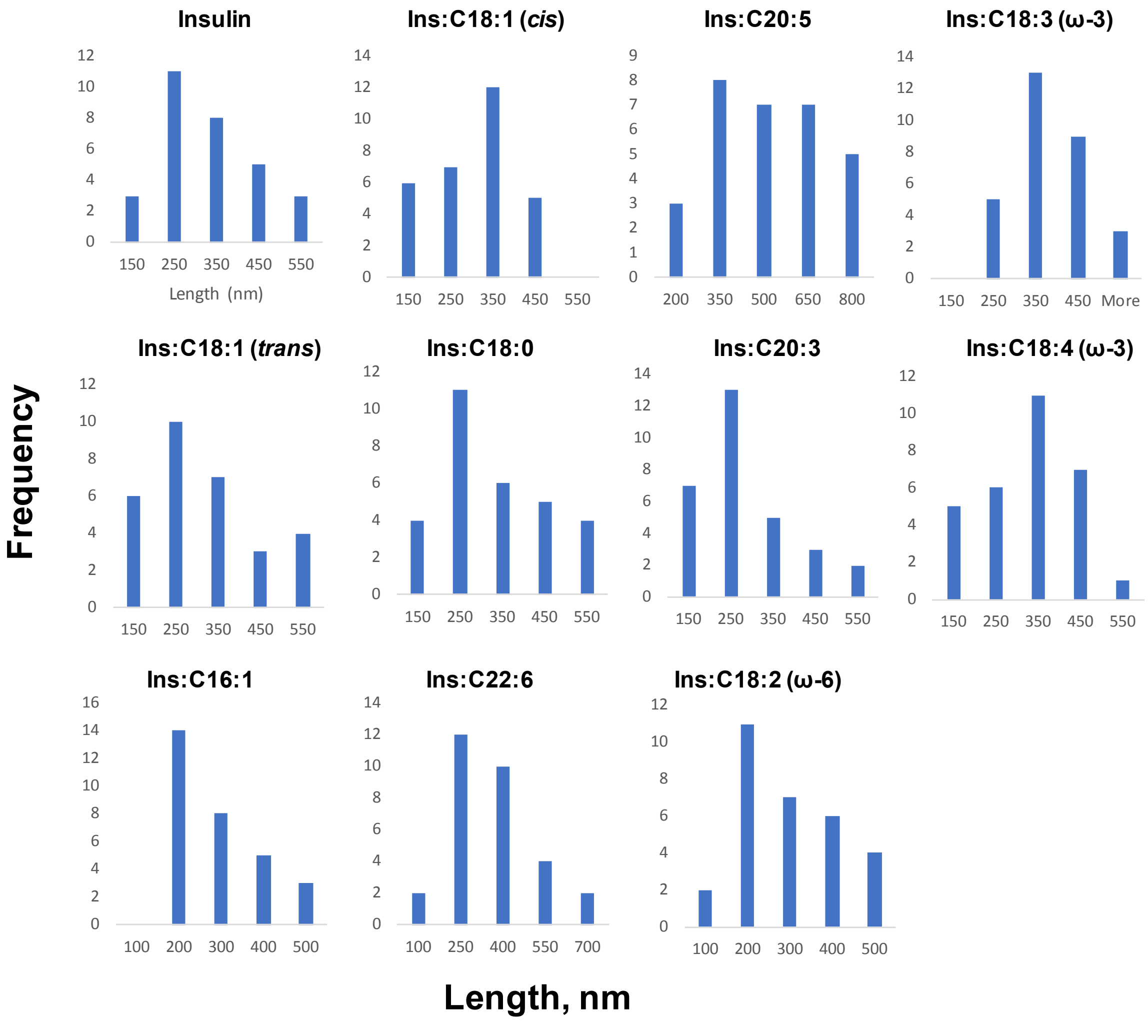

Figure S1. Histograms of length distributions of Ins fibrils and fibrils formed in the presence of FAs.

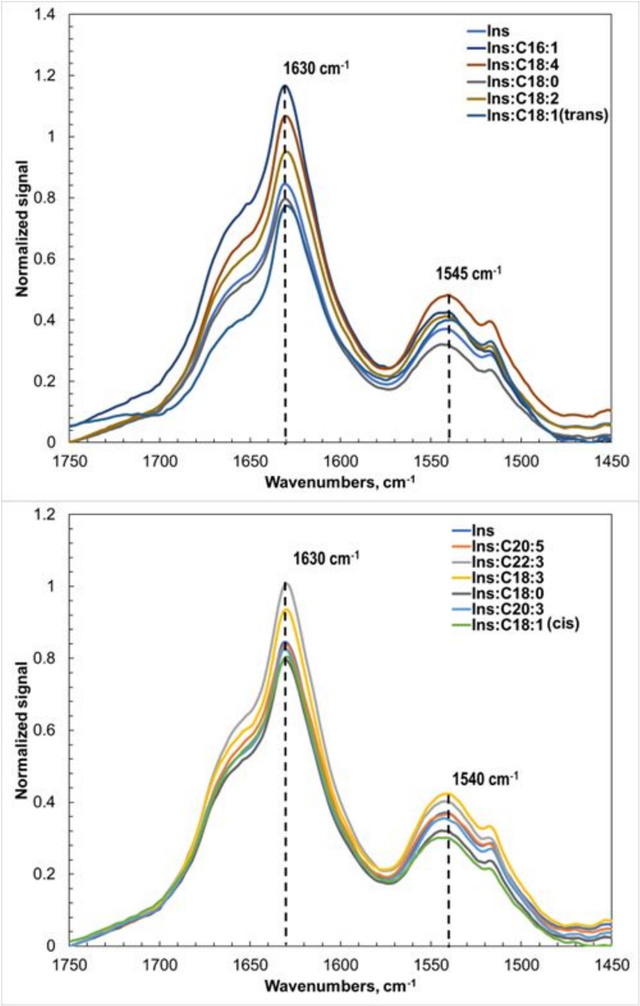

Figure S2. Elucidation of the secondary structure of insulin aggregates grown in the lipid-free environment (Ins) and the presence of LCUFAs and LCPUFAs. ATR-FTIR spectra of insulin aggregates grown in the lipid-free environment (Ins) and the presence of LCUFAs and LCPUFAs.

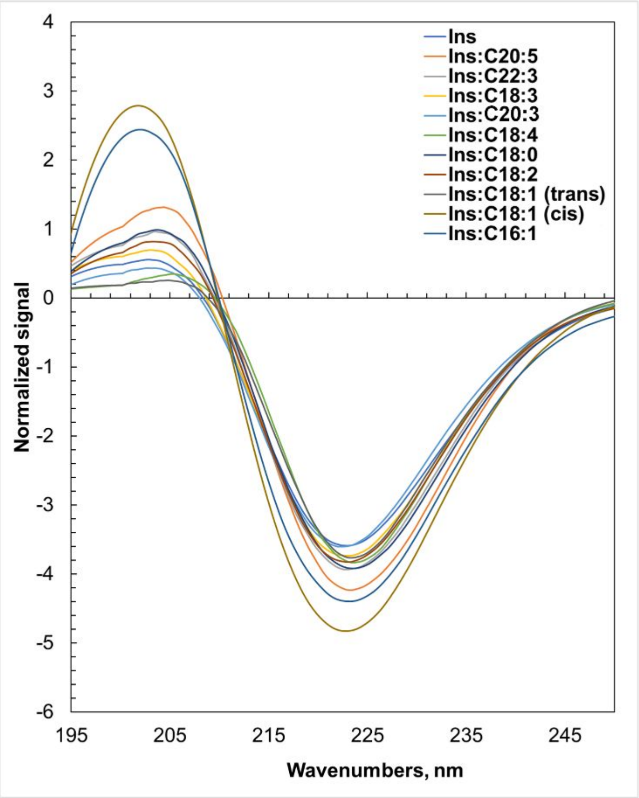

Figure S3. Analysis of the secondary structure of insulin aggregates grown in the lipid-free environment (Ins) and the presence of LCUFAs and LCPUFAs. CD spectra of insulin aggregates grown in the lipid-free environment (Ins) and the presence of LCUFAs and LCPUFAs.

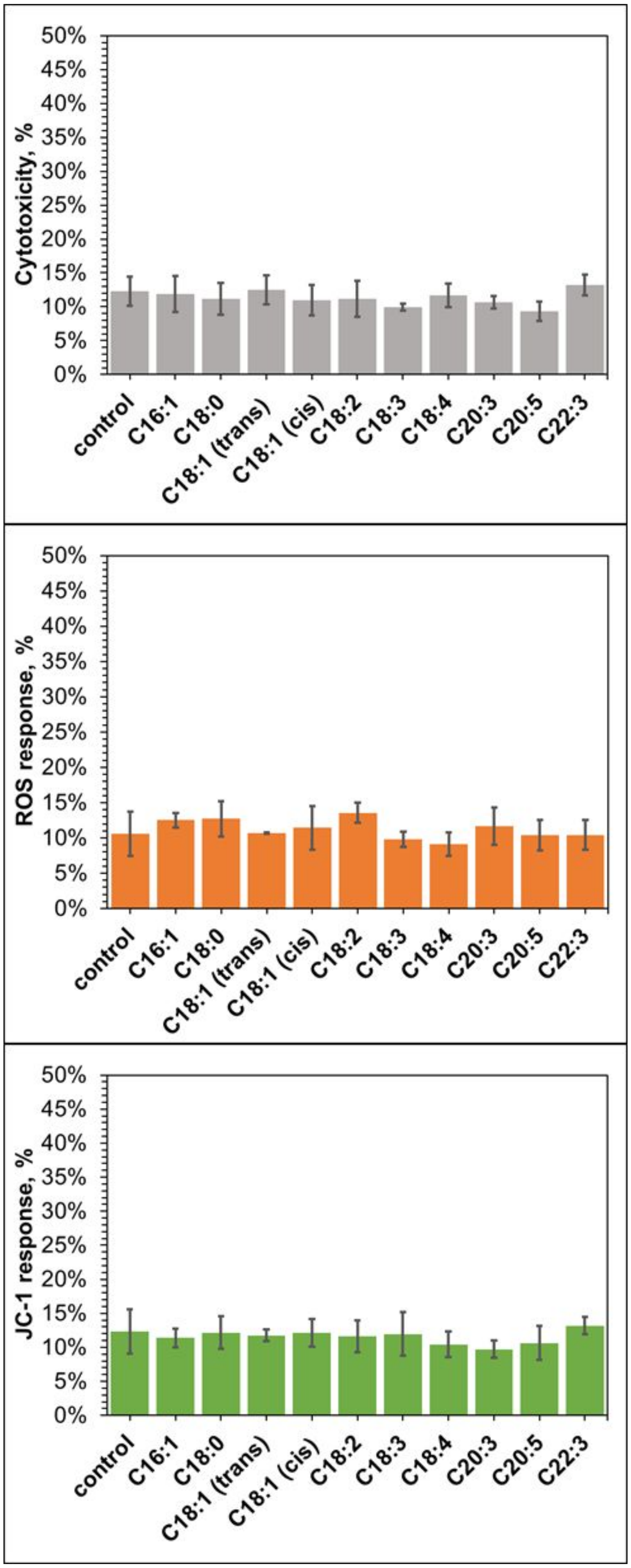

Figure S4. Histograms of LDH (top), ROS (middle) and JC-1 (bottom) assays of LCUFAs and LCPUFAs.
